# Origins of fast diffusion of water dimers on surfaces

**DOI:** 10.1038/s41467-020-15377-8

**Published:** 2020-04-03

**Authors:** Wei Fang, Ji Chen, Philipp Pedevilla, Xin-Zheng Li, Jeremy O. Richardson, Angelos Michaelides

**Affiliations:** 1 Thomas Young Centre, London Centre for Nanotechnology, University College London, London, WC1E 6BT UK; 2 Department of Chemistry, University College London, London, WC1E 6BT UK; 3 School of Physics and the Collaborative Innovation Center of Quantum Matters, Peking University, 100871 Beijing, China; 4 Laboratory of Physical Chemistry, ETH Zurich, CH-8093 Zurich, Switzerland; 5 Department of Physics and Astronomy, University College London, London, WC1E 6BT UK; 6 Max Planck Institute for Solid State Research, Heisenbergstrasse 1, 70569 Stuttgart, Germany; 7 State Key Laboratory for Artificial Microstructure and Mesoscopic Physics, and the Frontier Science Center for Nano-optoelectronics, Peking University, 100871 Beijing, China

**Keywords:** Reaction kinetics and dynamics, Density functional theory, Surfaces, interfaces and thin films

## Abstract

The diffusion of water molecules and clusters across the surfaces of materials is important to a wide range of processes. Interestingly, experiments have shown that on certain substrates, water dimers can diffuse more rapidly than water monomers. Whilst explanations for anomalously fast diffusion have been presented for specific systems, the general underlying physical principles are not yet established. We investigate this through a systematic ab initio study of water monomer and dimer diffusion on a range of surfaces. Calculations reveal different mechanisms for fast water dimer diffusion, which is found to be more widespread than previously anticipated. The key factors affecting diffusion are the balance of water-water versus water-surface bonding and the ease with which hydrogen-bond exchange can occur (either through a classical over-the-barrier process or through quantum-mechanical tunnelling). We anticipate that the insights gained will be useful for understanding future experiments on the diffusion and clustering of hydrogen-bonded adsorbates.

## Abstract (web summary)

The experimental observation that water dimers diffuse more rapidly than monomers across materials’ surfaces is yet to be clarified. Here the authors show by ab initio calculations classical and quantum mechanical mechanisms for faster water dimer diffusion on a broad range of metal and non-metal surfaces.

## Introduction

Interfacial water is ubiquitous and as such relevant to an almost endless list of processes and phenomena. Indeed interfacial water plays a central role in tackling some of society’s biggest challenges, such as global shortages of clean water, rising global temperatures, and the need for renewable energy. Improved understanding, particularly at the molecular level, offers the potential to make advances on each of these key global challenges. For these and other reasons, along with the fact that it is incredibly interesting scientifically, interfacial water has been extensively examined^1–3^, revealing complex behaviours of water clusters and overlayers on various surfaces^4^.

Of all the disciplines that have contributed towards improved understanding of interfacial water, studies of water on well-defined atomically flat surfaces under ultrahigh vacuum conditions have been particularly illuminating^1,2,5–10^. These studies have provided molecular-level understanding of the structure of water at the surfaces of inorganic materials (mainly metals and oxides). They have, for example, revealed an incredible richness in the variety of water overlayer structures that form. For specific systems, they have also revealed insight into the diffusion of water across surfaces^11–15^. For example, on the closed-packed Cu(111) surface low-temperature scanning tunnelling microscopy (STM) was recently used to measure the diffusion rate of individual water monomers and dimers^13^. Interestingly these measurements showed that the rates for water monomer and dimer diffusion were similar—an unexpected result given the heavier mass of the dimer and the stronger interaction of the dimer with the surface than the monomer. For adsorbate diffusion on crystalline materials there is a long-established rule-of-thumb which correlates the diffusion barrier with the strength of the adsorption bond. Thus, these measurements appear to be outliers of this rule-of-thumb. Moreover, it has been observed in earlier studies on Pd(111)^11^ and TiO_2_(110)^14^ that on both substrates water dimers diffuse even faster than water monomers. On Pd(111) it was suggested that facile hydrogen (H) bond donor–acceptor (DA) exchange—facilitated by quantum mechanical tunnelling—was key to the rapid motion of the dimers^16^. However, the general physical principles behind rapid water cluster diffusion remain to be understood. Obtaining this insight is important for understanding water flow across the surfaces of technologically relevant membrane materials^17,18^. Also the conclusion in the previous work was reached on the basis of rather crude theoretical estimates involving a one-dimensional WKB approximation. Whether this interpretation stands up to more rigorous theoretical analysis is unclear. While it is challenging for experiments to examine diffusion across many substrates, with computer simulation approaches broad ranging trend studies are much more feasible. In particular, density functional theory (DFT) has been successfully used to understand and interpret experimental data for water on numerous substrates^2,19–29^. Developments in van der Waals (vdW) inclusive density functionals have also helped to improve the accuracy of the predictions^30–32^. Nonetheless, most previous simulation studies on interfacial water diffusion have focused on specific individual systems, and the influence of nuclear quantum effects (zero-point motion and tunnelling) has generally not been taken into consideration.

Here we report the results of an extensive set of ab initio studies on a broad range of metal and non-metal surfaces, with the specific aim of understanding the general physical principles that facilitate rapid water dimer diffusion. The substrates considered include the close-packed (111) surfaces of Ag, Cu, Pt, Pd, Rh, and Al; the (100) surfaces of Pd and Al; the (100) surfaces of NaCl and MgO; and the (10$\overline{1}$0) surface of ZnO. These substrates are all of experimental interest and offer a diversity of surface structures and symmetries and have a wide range of physio-chemical properties (from a rather inert noble metal (Ag) to an ionic salt (NaCl) and a covalent oxide (ZnO)). On all of these substrates diffusion mechanisms and barriers for water monomers and dimers have been computed and when tunnelling is suspected to be important, path-integral theory has been used to describe the quantum nature of the nuclei^33,34^. In particular, semiclassical instanton theory^35,36^ is used to obtain the optimal tunnelling pathway and corresponding rate. The calculations reveal more cases where water dimers diffuse more rapidly than water monomers. The key to understanding the rich and diverse behaviour observed can be found in the balance between water–water and water–substrate bonding. Of all the systems considered, Pd(111) exhibits the most striking behaviour with water monomers predicted to diffuse faster at relatively high temperatures and water dimers predicted to diffuse faster at relatively low temperatures. This behaviour arises because tunnelling facilitates the exchange of the H-bond at low temperatures resulting in a non-Arrhenius temperature dependence of the dimer diffusion rate.

## Results

### Structures and mechanisms of water adsorption and diffusion

We first consider water monomer and dimer adsorption on the various substrates. The stable adsorption structures identified here agree with those observed in previous studies^20–22,25,27,37–41^. Key structural information is provided in Table 1. On all the metal surfaces studied, the water monomer adsorbs above a single metal atom at a top site, adopting an almost flat geometry. On the ionic crystals (NaCl and MgO), the water monomer also binds on top of a single metal atom in a flat-lying configuration, and on ZnO the water adsorbs in a surface “trench” on top of a Zn atom. The water dimer on the metal surfaces has a highly asymmetric geometry, with the H-bond donor molecule bonded relatively strongly to a top site (in a similar configuration to the monomer) and the H-bond acceptor  ~0.8 Å higher (~1.2 Å on Al) from the surface than the donor. On the non-metal surfaces the dimer structure is different, with both the H-bond donor and acceptor water molecules interacting strongly with the surface. The adsorption energies (*E*_ad_, definition see Table 1) of the water monomer and dimer are also reported in Table 1. The monomer adsorption energies on the different surfaces studied range from 0.3 to 1.0 eV, with Ag(111) having the weakest adsorption and ZnO(10$\overline{1}$0) having the strongest. The dimer adsorption energies range from 0.8 to 2.0 eV, again with Ag(111) having the weakest adsorption and ZnO(10$\overline{1}$0) having the strongest. In both cases, the dimer adsorption energies are larger than twice of that of the monomers.

**Table 1:** Water monomer and dimer adsorption geometries and energies on different surfaces.

Surface	*h* mono. (Å)	*h* D. (Å)	*h* A. (Å)	*d*_OO_ (Å)	${E}_{\,\text{ad}}^{\text{mono.}\,}$ (eV)	${E}_{\,\text{ad}}^{\text{dimer}\,}$ (eV)
Ag(111)	2.60	2.48	3.18	2.78	0.29	0.81
Cu(111)	2.37	2.23	3.07	2.75	0.32	0.90
Pt(111)	2.41	2.28	3.10	2.70	0.43	1.10
Pd(111)	2.38	2.27	2.97	2.73	0.46	1.12
Pd(100)	2.38	2.27	3.03	2.72	0.45	1.08
Rh(111)	2.32	2.23	3.02	2.71	0.53	1.22
Al(111)	2.20	2.08	3.27	2.69	0.45	1.15
Al(100)	2.15	2.01	3.27	2.64	0.40	1.13
NaCl(100)	2.33	2.34	2.36	3.18	0.45	0.97
MgO(100)	2.23	2.21	2.22	3.05	0.54	1.24
ZnO(10$\overline{1}$0)	1.83	1.87	1.88	3.00	0.98	2.05

*h* mono. is the height of the O atom in the water monomer to the surface (to the surface cation for ionic crystal surfaces). *h* D.(A.) is the height of the O atom in the donor (acceptor) water of the dimer to the surface. *d*_OO_ is the O–O distance in the adsorbed water dimer. The water adsorption energies are defined with respect to gas phase water monomers: ${E}_{\text{ad}}=-({E}_{n{\text{H}}_{2}\text{O/surf}}-{E}_{\text{surf}}-n\times {E}_{{\text{H}}_{2}\text{O}})$, where ${E}_{n{\text{H}}_{2}\text{O/surf}}$ is the total energy of the (H_2_O)_*n*_ adsorbed surface system, *E*_surf_ is the total energy of the relaxed bare surface slab and ${E}_{{\text{H}}_{2}\text{O}}$ is the total energy of the relaxed water monomer in the gas phase. Under this definition, *E*_ad_ is positive for all the systems studied. Note that water dissociation is not a main concern of this work for reasons briefly discussed in Supplementary Note 7.

**Fig. 1:**
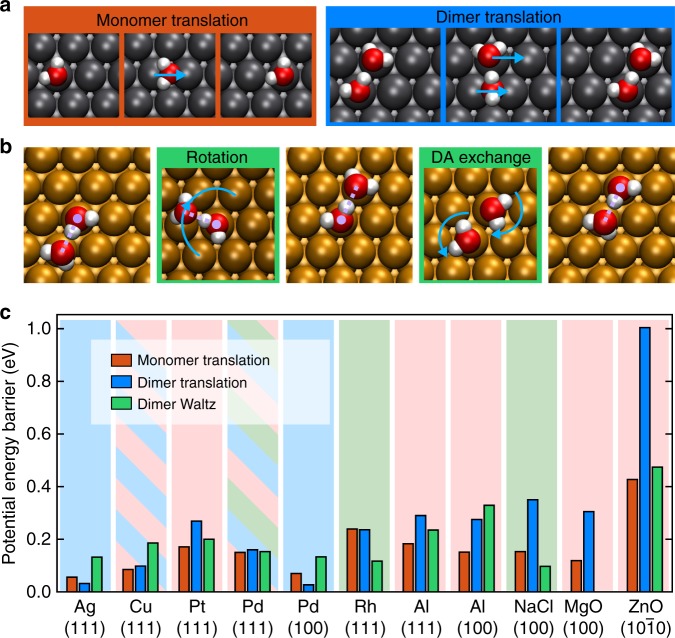
Water monomer and dimer diffusion mechanisms and barriers on a range of solid surfaces. **a** Top view of the water monomer and dimer translational diffusion pathway on Pd(111). **b** Top view of water dimer waltzing diffusion on an FCC(111) metal surface, showing the rotation and DA exchange steps. The violet dotted lines indicate the H-bonds in the dimer, with the large dot marking the H-bond donor. **c** Potential energy barriers for water monomer diffusion, dimer translation, and dimer waltzing diffusion on all the common surfaces studied. The background colour indicates the lowest barrier diffusion pathway on each surface, red: monomer diffusion; blue: dimer translation; green: dimer waltz. Multiple colours means that there are competing favourable diffusion mechanisms with barriers within 10 meV. All the barrier values are provided in the supporting information (Supplementary Table 2).

The monomer diffusion process on the metal surfaces is fairly straightforward involving a simple translational mechanism in which the water hops from one top site to another top site via a bridge site, which is the transition state (TS). The process is similar on all the metal surfaces examined, and a representative diffusion pathway is shown in Fig. 1a. There is a small orientational dependence of the water monomer hopping process but on the metal surfaces reorientation of the water monomer is also very facile^42^. Indeed, in general, there can be more than one pathway for a water monomer or dimer to translate on a surface, e.g. with a different water orientation at the TS, or moving along different lattice directions. We base our diffusion mechanism discussions on the minimum energy pathway with the lowest barrier, and examples of the other translation pathways are shown in Supplementary Note 5. For water monomer diffusion on non-metal surfaces we find that on NaCl and MgO surfaces, monomers diffuse to adjacent top sites of metal ions through a hopping mechanism over the bridge site, as reported in previous studies^15,41,43^; and on ZnO(10$\overline{1}$0), water monomers diffuse along the trenches on the surface, similar to what was found on TiO_2_^14^.

Water dimer diffusion, as with the monomer, can occur through a simple translation mechanism in which the dimer hops from one site to the next. Various possible dimer translation processes were considered on each surface and representative examples are shown in Fig. 1a. In this mechanism the H-bond donor and acceptor molecules diffuse from top sites to top sites via bridge sites in an almost concerted manner. The H-bond remains intact throughout the process and the relative heights of the molecules above the surface remain almost unchanged, regardless of whether the surface is metallic or not.

The second category of water dimer diffusion is a two-step mechanism involving rotation and a H-bond DA exchange, as illustrated in Fig. 1b with an example on an FCC(111) metal surface. For the specific example of dimer diffusion on Pd(111) this process has been dubbed a “waltz-like” diffusion mechanism^16^. We extensively investigated this type of diffusion pathway to find out whether it is preferable compared to the translational diffusion pathways and monomer diffusion. In the rotation step, the acceptor water rotates around an axis perpendicular to the surface at the donor water with the result that the acceptor water resides above a different surface atom. On metal surfaces, the acceptor water can almost freely rotate around the donor, meaning that the rotation step only needs to overcome a small energy barrier (e.g. 9 meV on Pd(111), 7 meV on Al(100)). STM experiments show that on a few metal surfaces, a water dimer has a six-fold symmetric flower-like shape or a round shape^5,13,44,45^, indicating that it is rapidly rotating even at low temperatures. On non-metal surfaces, in contrast, the rotation step is no longer free to happen and becomes the rate-limiting step. Relocating the acceptor water through rotation does not result in dimer diffusion itself. The second step, DA exchange, where the water molecules exchange their roles as H-bond donor and acceptor, is required to complete the diffusion process. On metal surfaces, the highly asymmetric water dimer adsorption geometry means that DA exchange is accompanied by a change in the height of the donor and acceptor molecules. A number of DA exchange processes are well known in gas phase studies of water clusters (see e.g. refs. ^46–48^). Here, there are three possible pathways for DA exchange: “twist–twist”, “twist–flip”, and “flip–flip”, leading to permutationally equivalent products. Illustrations and discussions of them are given in Supplementary Note 6. The “twist–twist” pathways give the lowest barriers on metals. On ZnO(10$\overline{1}$0) and NaCl(100), the DA exchange step has smaller barriers (0.2 and 0.01 eV, respectively) than the rotation step. A “twist–twist” pathway is favourable on NaCl(100), while on ZnO(10$\overline{1}$0) a “flip–flip” DA exchange is found perhaps because the water molecules are restricted in a trench-like surface structure.

The diffusion barriers computed for the various dimer diffusion mechanisms are shown in Fig. 1c. Some of the processes have been examined before with similar computational methods^16,37,41,49,50^ and whenever comparison to previous works is possible, the agreement is reasonable despite differences in exchange-correlation functional between this and earlier studies. Our predicted monomer diffusion barriers on Pd(111) and NaCl(100) are in quantitative agreement with previous experiments^11,15^, as are our monomer and dimer barriers on Cu(111)^13^. We highlight the most favourable diffusion mechanism on each surface in Fig. 1c with different colours in the background. Overall, these results reveal a rich variety of behaviour for water monomer and dimer diffusion on the surfaces considered. On some surfaces, the monomer diffusion barrier is the lowest (e.g. Al(111)), while on some others water dimer translation turns out to be favoured. For example, water dimers have a lower translational diffusion barrier than water monomers on Ag(111) and Pd(100). This prediction is in good agreement with experimental observations on Ag(111)^12,51^. Then, some surfaces act like a “stage” hosting water dimer “waltzing” most favourably, e.g. Rh(111) and NaCl(100), while on some other surfaces, a number of different diffusion mechanisms are in close competition, e.g. Pd(111). At first glance, it seems that water monomer and dimer diffusion on surfaces needs to be understood on a surface by surface basis, since the favourable diffusion mechanism varies from one surface to the next. However, close inspection reveals several interesting trends, and we discuss them in detail in the following sections, starting with translational diffusion and then the waltz-like diffusion.

### Analysis of water monomer and dimer diffusion barriers

**Fig. 2:**
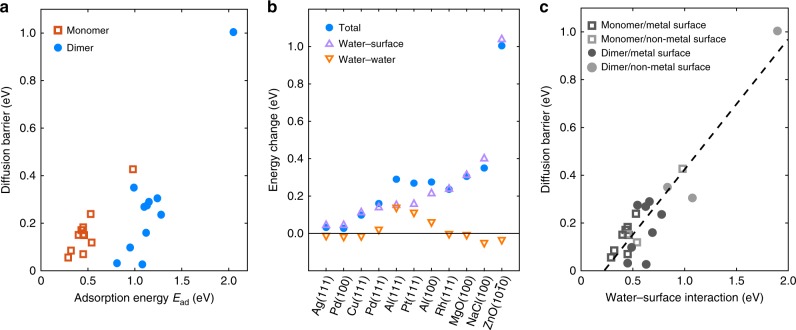
Trends in water monomer and dimer translational diffusion barriers. **a** Correlation between water monomer and dimer translational diffusion barriers and the adsorption energy across a range of surfaces. A linear regression of the monomer barriers gives *R*^2^ = 0.83, however, taking both the monomer and dimer into account, a linear regression gives *R*^2^ = 0.54. **b** Decomposition of the energy change between the TS and the initial state for water dimer translation, showing the barriers predominately arise from water–surface interactions. **c** Correlation between water monomer and dimer translational diffusion barriers and water–surface interaction strength across a range of surfaces. The dashed line is a linear regression of the data points (*R*^2^ = 0.85).

We start by examining the trend in water monomer diffusion barriers, the least complex of all. Monomer diffusion barriers show a good correlation with the water monomer adsorption energy (Fig. 2a). This behaviour is expected, obeying the rule-of-thumb that relates the diffusion barrier with the adsorption energy^52^. In this case, linear regression suggests that the barrier depends linearly on approximately half the adsorption energy.

Next we examine the relation between the dimer translation barriers and the adsorption energy. We note that the adsorption–diffusion rule^52^ was established for single atom and molecule diffusion on transition metal surfaces, and it remains an open question whether that conclusion applies to dimers and non-metal surfaces or not. The dimer barriers more or less follow the adsorption–diffusion rule. However, if one looks at the monomer and dimer translation barriers together in Fig. 2a, the correlation becomes much weaker. On quite a few metal surfaces, the dimer translation barrier is comparable to or even smaller than the monomer barrier, despite the fact that the dimer adsorption energy is at least twice the monomer adsorption energy, breaking the correlation. We also tested the correlation using different definitions of the dimer adsorption energy (see Supplementary Fig. 4), in the best case we found *R*^2^ = 0.68, which is still considerably worse than the correlation for the monomer.

To understand the physical origin of the diffusion barriers, we decompose the water dimer adsorption energy into the following two terms:

1 $${E}_{\text{ad}}({\text{dimer}})\to {E}_{\text{water-surf}}+{E}_{\text{water-water}}$$

in which *E*_water–surf_ = *E*_ad_(donor) + *E*_ad_(acceptor) characterises the water–surface interaction (*E*_ad_ is defined in Table 1); *E*_water–water_ = *E*_ad_(dimer) − *E*_water–surf_, is the water–water interaction and it predominately characterises the H-bond interaction at the adsorbate state. Detailed results of the decomposition analysis are given in Supplementary Note 3, and we note that decompositions schemes for estimating specific bond strengths are to some extent arbitrary. Our analysis reveals that strong water–water interactions are present in the highly asymmetric water dimers on metal surfaces with energies of  ~0.4 eV (Supplementary Table 3), much larger than that in gas phase water dimer (~0.2 eV). This is a result of a cooperative enhancement of the H-bond strength in the presence of the surface^53^. This phenomenon is well-known and is also indicated by a clear shortening of the H-bond compared to the gas phase (Table 1). For water dimers on ionic and covalent crystal surfaces, however, the water–water interaction strengths are slightly weaker than the gas phase water dimer and the H-bonds are longer.

Previously, we found that on Cu(111)^13^, the water–water interaction does not contribute much to the barrier. We found that this is also true for most of the surfaces studied here (both metal and non-metal), as shown in Fig. 2b. Therefore, one can infer that the main contribution to the translational diffusion barrier comes from the water–surface interaction (Eq. (1)), instead of the adsorption energy. We can use this to understand the dimer translation barriers on metal. One consequence of the strong H-bond interaction within the dimer on metals is that the H-bond acceptor water interacts relatively weakly with the substrate. Because of this, the water–surface interactions of the dimer on metals are generally smaller than those on other surfaces when the donor and acceptor sit at similar heights. The reduced water–surface interactions may induce smaller dimer diffusion barriers that are comparable to monomer barriers. Furthermore, we can correlate the water monomer and dimer translational diffusion barriers with the water–surface interactions (Fig. 2c). We found a clear trend showing that stronger water-surface interactions give larger diffusion barriers (linearly dependent on  ~0.5 water–surface interaction), regardless of whether the surface is metallic, ionic, or covalent. This simple correlation could be useful for naive comparisons (perhaps even predictions) of water translational diffusion barriers on surfaces in general. It might also be useful in understanding diffusion of other H-bonded adsorbate clusters.

**Fig. 3:**
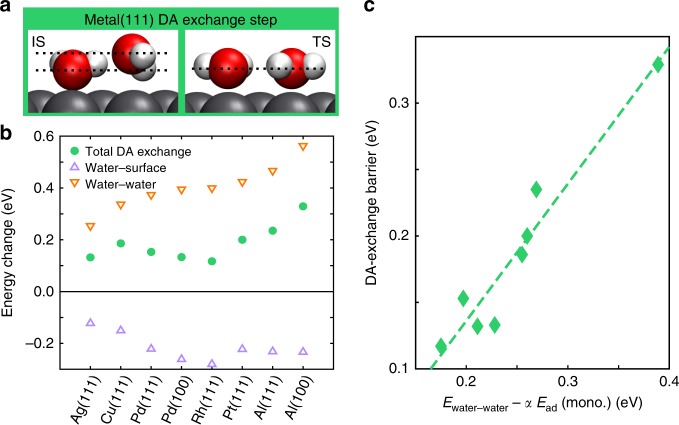
Trends in water dimer waltz diffusion on metals. **a** Side view of the water dimer of the initial state (IS) and transition state (TS) of the DA exchange process on Pd(111), viewed from the [$\overline{2}$11] direction. The dotted lines indicate the heights of the two water molecules. **b** Decomposition of the energy change between the TS and the IS for water dimer DA exchange on metal surfaces. **c** Correlation between the potential energy barrier of the DA exchange step on metal surfaces and a characterisation quantity defined by the water–water interaction (Eq. (1)) and water monomer adsorption energy, with *α* = 0.5. The dashed line is a linear regression of the data points (*R*^2^ = 0.93).

Next, we analyse the trends in the water dimer waltzing diffusion. In this two-step mechanism, the DA exchange is the rate-limiting step on metal surfaces. Comparing the TS geometry to the adsorption state (see Fig. 3a), one can see that the acceptor water comes down to the same height as the donor water at the TS. This indicates that water monomer adsorption interaction plays a role: the stronger the adsorption, the more it stabilises the TS. The H-bond is also opened up at the TS. This means that the water–water interaction is another important factor to this process: the stronger the H-bond, the harder it is to break. Indeed, decomposition of the DA exchange barriers (Fig. 3b) shows that the main contribution to the DA exchange barrier comes from water–water interactions. The water–surface interaction plays a secondary (while also important) role. This finding helps us understand how the DA exchange barrier changes upon moving from one metal surface to the next. For example, on Pd(111) and Pt(111), water monomers have similar adsorption energies while the water dimer has a stronger H-bond on Pt (as indicated by the O–O distance in Table 1), hence the DA exchange barrier on Pt(111) is higher than on Pd(111). On Al(100), the DA exchange barrier is the highest of all because it has the strongest water–water interaction. The DA exchange barrier decreases going from Cu(111) to Pd(111) to Rh(111) mainly because the water–surface interaction increase from Cu to Pd to Rh. Combining the two factors, we find that the DA exchange barrier is well approximated by the quantity: *E*_water–water_ − *αE*_ad_(mono.) + *s*, as shown in Fig. 3c, where *α* = 0.5 and the shift *s* = −0.07 eV.

This relation can help us predict the DA exchange barrier on new surfaces simply with two basic water dimer adsorption properties, which could be accessible experimentally^54^. Furthermore, it determines when the dimer waltz mechanism becomes favourable compared to the monomer diffusion on metal surfaces. As we have shown in Fig. 2a, monomer barriers tend to increase with water monomer adsorption energy. DA exchange barriers, as discussed above, tend to decrease with monomer adsorption energy. Therefore, it seems that dimer waltzing becomes favourable on metals with relatively large adsorption energy. Rh(111) is a prime example of this, which could have interesting implications for experiments. We expect that in a STM experiment on Rh(111), one may observe the water monomer diffusing slowly, but once two monomers join, a single rapid-diffusing dot could be observed, similar to ref. ^11^.

On non-metal surfaces, the dimer rotation has a significant barrier and becomes the rate-determining step of the waltz mechanism. One can see from side views of the rotation TSs on NaCl(100) and ZnO(10$\overline{1}$0) (Supplementary Fig. 6), that the acceptor water is lifted up from the surface during this process. Since both molecules in the dimer adsorb at the same height on the non-metal surfaces examined, energy is required to move the water higher above the surface. In contrast, on metal surfaces, since the asymmetric adsorption geometry is preferred, the rotation step is almost free energetically. On ZnO(10$\overline{1}$0), the barrier of the rotation step (which determines the waltz diffusion rate) is lower than the dimer translation barrier, albeit still higher than the monomer barrier. However, there are cases where the waltz mechanism becomes favourable: a previous study showed (with DFT) that it is the mechanism behind the fast water dimer diffusion observed in experiments on rutile TiO_2_^14^. On NaCl(100), the rotation step has a slightly lower potential energy barrier than monomer translation. Therefore, we expect that there could be an experimentally interesting behaviour on NaCl(100): when two water molecules join into a water dimer, it would diffuse faster; and when the water dimer breaks apart, both fragments move slower than the cluster.

### Nuclear quantum effects in water dimer diffusion

The water dimer waltz mechanism on metal surfaces is rate-limited by the DA exchange step, in which the H atoms rearrange. Due to the light masses involved, NQEs are expected to play a role, especially at the low temperatures at which STM experiments are conducted. A first step to estimate the importance of NQEs is to look at the zero point energy (ZPE) and the crossover temperature to tunnelling ${T}_{\text{c}}=\frac{\hslash {\omega }^{\ddagger }}{2\pi {k}_{\text{B}}}$, where *ω*^‡^ is the magnitude of the imaginary frequency at the TS^55,56^. *T*_c_ is the temperature at which the instanton, which uniquely defines the optimal tunnelling pathway^35,36^, starts to delocalise across the barrier. Hence it is often used as an indication of a temperature region near and below which tunnelling should be considered in a given reaction process. In Table 2, we show the harmonic ZPE corrections and *T*_c_ for water diffusion on all transition metal surfaces, obtained using harmonic frequencies calculated from the ab initio simulations. The DA exchange step for the H_2_O dimers has a *T*_c_ of  ~40 K, meaning that the experimental temperature (40 K in ref. ^11^) is close to the crossover temperature on all these surfaces. For D_2_O dimers, the *T*_c_ is lower (30 K). *T*_c_ for translational diffusion processes are around 20 K or lower.

**Table 2:** *T*_c_ and ZPE corrections for water monomer and dimer diffusion on metal (111) surfaces.

	DA exchange step			Dimer trans.		Mono. trans.	
	*T*_c_ (K)	${T}_{\,\text{c}}^{\text{D}\,}$ (K)	ZPE (eV)	*T*_c_ (K)	ZPE (eV)	*T*_c_ (K)	ZPE (eV)
Ag	45	32	−0.020 (15%)	9	−0.014 (44%)	11	−0.025 (45%)
Cu	44	32	−0.026 (14%)	12	−0.021 (21%)	17	−0.025 (29%)
Pt	41	30	−0.020 (10%)	22	−0.040 (15%)	19	−0.044 (26%)
Pd	39	29	−0.017 (11%)	17	−0.025 (16%)	21	−0.030 (20%)
Rh	40	29	−0.019 (16%)	22	−0.028 (12%)	23	−0.039 (16%)

*T*_c_'s of D_2_O (${T}_{\,\text{c}}^{\text{D}\,}$) for DA exchange are also given. The numbers in the brackets show the percentage harmonic ZPE correction with respect to the potential energy barrier.

**Fig. 4:**
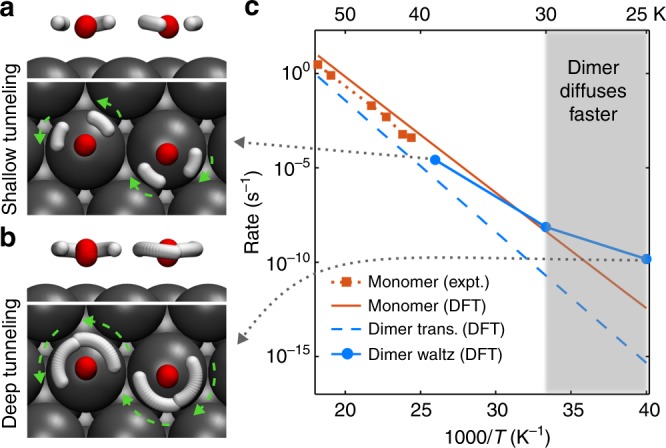
Tunnelling in water dimer DA exchange step. **a** Side and top views of the H_2_O instanton trajectory at 38.5 K with 32 beads. **b** Side and top views of the H_2_O instanton trajectory at 25 K with 64 beads. **c** Diffusion rates versus temperature for the water monomer and dimer diffusion processes. Experimental rates from ref. ^11^ are shown for comparison. The shaded area marks the temperature regime where the dimer diffusion becomes faster than the monomer diffusion.

We chose to study the Pd(111) surface with the instanton theory as an example, because water diffusion on this surface has been measured experimentally^11^. Also there are closely competing pathways on this surface, suggesting that quantum tunnelling could lead to a change in the diffusion mechanism. The instanton theory accounts for both ZPE effects and quantum tunnelling, while the latter is absent from simple ZPE corrections. Instanton pathways, which characterise the optimal tunnelling path, are shown in Fig. 4, and indeed show that in the DA exchange step below 40 K the two waters rotate by tunnelling to rearrange the H-bond. As the temperature decreases, the tunnelling path gradually becomes more delocalised, showing a smooth transition from shallow to deep tunnelling. Interestingly, the instantons also reveal that H tunnelling happens in conjunction with tunnelling of the heavier O atoms. We show the O tunnelling distance, defined as the delocalisation length of an O in the instanton, at different temperatures in Table 3. This finding is a departure from the previously established picture where two water molecules first come to the same height and then from there H tunnelling happens^16^, and shows that reduced dimensionality treatments of the dynamics are inadequate. Experimental evidence of contributions from O tunnelling has been reported in the literature in similar processes, such as the O^16^/O^18^ isotope effect on tunnelling splitting in the water hexamer prism^48^.

**Table 3:** Characteristic quantities of tunnelling calculated from the instantons at different temperatures.

Temperature (K)	O tunnelling distance (Å)	Activation energy reduction (eV)
38.5	0.05 (7%)	0.012 (8%)
30	0.15 (20%)	0.018 (12%)
25	0.22 (28%)	0.030 (20%)

The O tunnelling distance on Pd(111) is characterised by the delocalisation length of the instanton trajectories along the direction perpendicular to the surface. The brackets show the percentage of the O tunnelling distance with respect to the O height change of the DA exchange process. The activation energy reduction due to quantum tunnelling is defined as: $\frac{1}{\beta }\rm{log}\,\frac{{k}_{\text{inst}}}{{k}_{\text{TST}}}$, in which *k*_inst_ is given in Eq. (2), *k*_TST_ is the Eyring TST rate^57^ computed with *N*-bead ring-polymer partition functions^73^. This reduction can be viewed as “on top of” the ZPE correction. The brackets show the percentage reduction with respect to the potential energy barrier. At 25 K, the effective barrier reduction obtained using 64 beads is also 0.030 eV, implying that convergence with *N* has been achieved.

We calculated the water diffusion rates on Pd(111) as a function of temperature for the water dimer DA exchange and the monomer and dimer translational diffusion processes (Fig. 4). The translational diffusion processes are above their crossover temperatures, therefore the Eyring transition state theory (TST) rates^57^ are presented. The monomer diffusion rates are in good agreement with experiment^11^. The rate of water dimer waltzing exceeds the rate of water monomer diffusion and becomes the fastest water diffusion mechanism on Pd(111) at low temperature, as in the experiment. This suggests that quantum tunnelling is another mechanism to enable the water dimer to diffuse faster than the water monomer (at low temperatures). Sensitivity of the diffusion rates to the uncertainties in the DFT barriers and the impact of other factors are discussed in Supplementary Notes 9 and 10. Extending this finding, we expect that on Rh(111), the water dimer diffusion rate will display non-Arrhenius behaviour at low temperatures, which could be probed in future experiments.

By comparing the instanton rates and the Eyring TST rates, one can see that quantum tunnelling can reduce the effective activation energy of DA exchange on metals by up to 30 meV in the temperature range of 40–25 K (Table 3). Thus tunnelling makes the rate a million times faster at 25 K. If O were classical (estimated by reoptimising the instanton with the O mass increased by 10 fold), the dimer diffusion would be almost two orders of magnitude slower at 25 K (see Supplementary Note 8), although the main tunnelling contribution still comes from H atoms. This means that if the energy barrier gap between the water dimer DA exchange step and monomer diffusion is small, quantum tunnelling can be significant enough to make the water dimer waltz faster than the monomer at low temperatures. For example, it might be possible that tunnelling can also make the water dimer diffusion rate on Pt(111) or Al(111) comparable to the monomer rate at low temperatures, however the overall diffusion rate could be too slow to be observed in experiments.

## Discussion

By extensively studying water monomer and dimer diffusion on a range of common surfaces, we have shown that diffusion of dimers can exceed that of monomers on several substrates and that rapid dimer diffusion is thus more widespread than previously anticipated. In the classical regime, the H-bond enables dimers to diffuse faster than monomers by causing a reduction in the dimer translational barrier, or by enabling waltz-like diffusion mechanisms. By analysing the seemingly random diffusion barriers, we identified clear trends that the translation barriers correlate with the water–surface interaction energy, and the DA exchange barriers correlate with a linear combination of the H-bond strength and the monomer adsorption energy. The second scenario involves quantum tunnelling (assisted by heavy atom tunnelling). Tunnelling in the DA exchange step of water dimer waltzing on metal surfaces induces a non-Arrhenius temperature dependence of the dimer diffusion rate. This can result in the dimer diffusion becoming faster than the monomer only when temperatures is lowered, e.g. on Pd(111) where the monomer and dimer diffusion barriers are similar. This work provides insights for predicting water monomer and dimer diffusion behaviour on general surfaces, as well as guidance for future experiments of water dynamics on surfaces. Extending these findings, we think that the diffusion of other H-bonded clusters on surfaces^58,59^ could display peculiar behaviours as a result of strong H-bond, diffusion mechanisms via H-bond rearrangements, and NQEs.

## Methods

### DFT calculations

Our density-functional theory calculations were carried out using the Vienna ab-initio simulation package (VASP)^60^ with the optB88-vdW functional^61^, which provides an accurate description of H-bonding in water^62^ and has been widely used for studying water on surfaces^15,27,28,41^. The lattice parameters of a variety of solids predicted by the optB88-vdW functional are also in good agreement with the experimental lattice parameters^63^. Previous studies have shown that vdW interactions are important to water adsorption on surfaces, by increasing the adsorption energy by more than 0.1 eV per water, while the binding geometries are barely affected by the inclusion of vdW interactions^27,40,41,64–66^. A plane-wave cutoff of 600 eV was used throughout. The metal surfaces were represented using a four-layer-thick slab in a 4 × 4 unit cell with a 3 × 3 × 1 *k*-point mesh. NaCl(100) and MgO(100) was represented using a three-layer-thick slab in a 2 × 2 unit cell with a 2 × 2 × 1 and 3 × 3 × 1 *k*-point mesh, respectively. ZnO(10$\overline{1}$0) was represented using a three-layer-thick slab in a 2 × 3 unit cell with a 2 × 2 × 1 *k*-point mesh. A vacuum of at least 11 Å was placed above each surface and a dipole correction^67^ was also applied. Tests on the computational step up and functional dependence (including tests on the self-interaction error) are given in Supplementary Note 1. The climbing image nudged elastic band (cNEB) method^68^ was used to obtain the potential energy barriers and minimal energy pathways for water monomer and dimer diffusion. The force convergence criteria for the geometry optimisations and cNEB calculations is 0.01 eV Å^−1^ (for the cNEB calculations on non-metal surfaces the convergence criteria is 0.02 eV Å^−1^ instead). Tests show that on a metal surface, using a flexible or fixed surface were found to have a negligible effect on the cNEB barriers (for example 4 meV for the DA exchange step of dimer waltz on Pd(111)), because there is little surface deformation when a water monomer or dimer is adsorbed, hence fixed surfaces were used. We also note that previous studies find that on metal surfaces, surface dynamics only play a minor role for hydrogen diffusion^69^. For the ionic and covalent crystal surfaces, we discuss in Supplementary Note 4 that the flexibility of the surface has only a quantitative but not a qualitative impact on the results in this work. The diffusion barriers generally increase by 10–30%, yet the adsorption energies also increase by  ~10%, which is in line with the idea that adsorbates that adsorb stronger diffuse slower. Also, surface flexibility does not qualitatively change the water–water interaction. Hence we fixed the surfaces for simplicity.

### Instanton rate calculations

Tunnelling rates and pathways were computed with ring-polymer instanton rate theory^36,70–73^, a quantum TST-like method applicable to ab initio studies of NQEs in reactions and diffusion processes^72–75^. The instanton rate is given by

2 $${k}_{\text{inst}}(\beta ;N)={A}_{\text{inst}}(\beta ;N)\ {{\rm{e}}}^{-S[{\bf{x}}]/\hslash },$$

in which **x** is the *N*-bead ring-polymer instanton with imaginary time *βℏ* (*β* = 1∕*k*_B_*T*), *S* is its Euclidean action, and *A*_inst_ is a measure of fluctuations around the instanton path^36,73^. The multidimensional instantons were obtained via first-order saddle point optimisations of the effective potential *S*[**x**] ∕ (*βℏ*) at different temperatures, with the total force converged to below 0.02 eV Å^−1^. The potential energy surface was calculated on-the-fly with DFT, performed using a python wrapper based on the atomic simulation environment (ASE) library^76^. The instanton rates were extrapolated to the infinite bead limit by multiplying the ratio of the infinite bead to *N*-bead Eyring TST rates^73^. Thirty-two beads were used to represent the instanton, and we demonstrate in Table 3 that this is converged at the lowest temperature considered.

## Supplementary information

Supplementary Information

## Data Availability

All structures computed are provided as a Source Data file.

## Source data

Source Data

## Code availability

The code for performing instanton calculations is available from the authors on request.
