# Supplementary material for: Origins of fast diffusion of water dimers on surfaces
Source: Nat Commun. 2020 Apr 3;11:1689. doi: 10.1038/s41467-020-15377-8 (PMC7125088; doi:10.1038/s41467-020-15377-8)
Supplement: Supplementary file 1 — Supplementary Information [file 41467_2020_15377_MOESM1_ESM.pdf]

Supplementary information

Origins of fast diffusion of water dimers on surfaces

Fang et al.

SUPPLEMENTARY NOTE 1. COMPUTATIONAL SETUP OF THE DFT CALCULATIONS

The convergence of the water monomer and dimer diffusion barriers with respect to the unit cell size have been tested. This test serves a similar purpose as testing the convergence with respect to K-points, here it is more suitable as we are modelling isolated molecules on infinite surfaces. A 3×3 cell (smallest O-O distance between periodic images is 6.4 Å for the water dimer), 4×4 cell (smallest O-O distance between periodic images is 8.5 Å for the water dimer), up to a 9×9 cell (smallest O-O distance between periodic images is 22.5 Å for the water dimer) have been considered on a transition metal surface. One can see

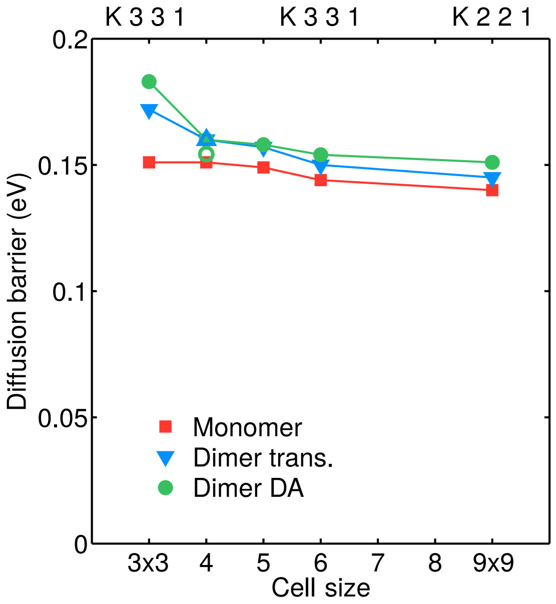

Supplementary Figure 1. Convergence of the water monomer and dimer diffusion barriers on Pd(111) with respect to the unit cell size. The labels "dimer trans." stands for water dimer translational diffusion and "dimer DA" stands for the dimer DA exchange step in the waltz diffusion mechanism. The water geometries are kept fixed at the optimised geometry in the 3×3 cell. The open triangle marks the geometry optimised dimer translation barrier and the open circle marks the geometry optimised dimer DA exchange barrier in the 4×4 cell. The K-points used for each cell size are shown on the top.

from Supplementary Figure 1 that on Pd(111), a 3×3 cell gives reasonable water diffusion barriers with an over estimation within 30 meV, and a 4×4 cell reduces the error to 10

meV compared to the very large 9×9 cell. For the other metal (111) surfaces, the smallest lattice constant is only 8% smaller than Pd, hence we expect the convergence with respect to the cell size is similar across all the metal surfaces studied. On NaCl(100), MgO(100), and ZnO($10\bar{1}0$), the shortest O-O distances are 8.5 Å, 6.1 Å, and 6.9 Å respectively. Due to that the dimer diffusion barriers on non-metal surfaces are much higher than on metals, the relative impact caused by the interaction between periodic water images should be less significant.

We also tested the convergence of the diffusion barrier with respect to the number of layers on two examples. On Pd(111), the monomer diffusion barrier is only 4 meV different between a 4 layer (6.8 Å thick) and a 9 layer slab (13.1 Å thick), suggesting that a 4 layer slab is converged. On ZnO(1010), the dimer translation barriers on a 3 layer slab (6.6 Å thick) and a 4 layer slab (9.5 Å thick) differ by only 3 meV.

The sensitivity of the water monomer and dimer diffusion barriers to DFT exchange-correlation functional has also been tested on Pd(111). Three other van der Waals inclusive functionals (optB86b-vdW,[1] revPBE-D3,[2,3] and TPSS-D3[3,4]) are tested, and the results are compared with the optB88-vdW[5] functional used in the main text. These functionals were chosen because they predict accurate lattice parameters for Pd within 2% error with the experimental value of 3.88 Å, and have shown excellent performances for gas phase water clusters.[6–8] We first relaxed the lattice and surface, and then performed climbing image nudged elastic band (cNEB) calculations for each functional to obtain the diffusion barriers. The results are shown in Supplementary Table 1, one can see that for the water monomer

DFT functional	Water monomer	Dimer translation	Dimer DA exchange
optB88-vdW[5]	0.150	0.160	0.153
optB86b-vdW[1]	0.159	0.172	0.158
revPBE-D3[2,3]	0.118	0.166	0.176
TPSS-D3[3,4]	0.147	0.169	0.176

Supplementary Table 1. Comparison of the water monomer and dimer diffusion barriers (units in eV) on Pd(111) calculated using different functionals. A 4×4 cell with 4 layers and 600 eV plane-wave cutoff is used.

diffusion, three functionals agreed very well, predicting the barrier to be ~0.15 eV. Since

we show in the main text (Supplementary Figure 4(c)) that the monomer diffusion rate calculated using the optB88-vdW functional agree well with experimental rates,[9] we think the revPBE-D3 barrier is less reliable for this system. The dimer diffusion barriers agree within ∼0.02 eV for all the functionals tested. Hence we conclude that the diffusion barriers are not very sensitive to the DFT functional among good options for this system.

Finally, we consider self interaction errors by testing a hybrid functional for all the water diffusion barriers on NaCl(100). The HSE06[10] functional along with the D3 dispersion correction[3] is used, and we calculate the diffusion barriers based on the optimised initial state and transition state geometries with the optB88-vdW functional. We found that the hybrid functional predicts that the water monomer diffusion barrier, water dimer transition barrier, and the water dimer waltz diffusion barriers are 0.141 eV, 0.325 eV, and 0.080 eV respectively. These barrier values agree well with the barriers predicted by the optB88-vdW functional, which are 0.153 eV, 0.350 eV, and 0.098 eV respectively. The errors are less than 0.025 eV. In fact, DFT performs quite well for diffusion and H-bond rearrangement processes, unlike in covalent bond breaking processes where self interaction can be a severe problem. This has been shown in previous studies through comparison to higher levels of theory in the gas phase and to experiment on surfaces.[6,11–13] We also note that our water monomer adsorption energy on NaCl(100) obtained with the optB88-vdW functional (0.45 eV) is also in excellent agreement with a previous benchmark value (0.487 ± 0.06 eV) calculated with embedded CCSD(T).[14]

SUPPLEMENTARY NOTE 2. WATER MONOMER AND DIMER DIFFUSION BARRIER DATA AND PATHWAY GEOMETRIES

The water monomer and dimer diffusion pathway geometries on metal surfaces are shown in the main text, here we show the pathways on non-metal surfaces (Supplementary Figure 2). Water monomer and dimer translational diffusion processes on MgO(100) are similar as on NaCl(100). The water dimer translations along the H-bond direction. On ZnO($10\bar{1}0$), water monomer and dimers move along a trench on the surface, similar to on rutile TiO_2(110).[15] For the waltz diffusion mechanism, in the first step, the acceptor water lifts its O atom up from the surface with the help of the donor water, rolls-over, then lands on top of a different surface atom. This is the bottleneck step on non-metal surfaces. After

	monomer trans	dimer trans	dimer waltz
Ag(111)	0.056	0.032	0.132
Cu(111)	0.085	0.098	0.186
Pt(111)	0.171	0.269	0.200
Pd(111)	0.150	0.160	0.153
Pd(100)	0.070	0.027	0.133
Rh(111)	0.239	0.236	0.117
Al(111)	0.183	0.290	0.235
Al(100)	0.151	0.275	0.329
NaCl(100)	0.153	0.350	0.097
MgO(100)	0.119	0.305	-
ZnO($10\bar{1}0$)	0.427	1.004	0.474

Supplementary Table 2. Water monomer and dimer diffusion barrier data (units in eV).

this step, the water dimer is displaced by one lattice, but the H-bond direction is the opposite as the initial state. Hence, the second step, where the H-bond donor and acceptor exchange roles is needed to complete the diffusion process.

SUPPLEMENTARY NOTE 3. DECOMPOSITION OF THE WATER DIMER ADSORPTION ENERGY

The adsorption energies (E_{ad}) of the water monomer and dimer are defined as:

$$E_{\text{ad}} = -(E_{n\text{H}_2\text{O/surf}} - E_{\text{surf}} - n \times E_{\text{H}_2\text{O}}), \tag{1}$$

where $E_{n\text{H}_2\text{O/surf}}$ is the total energy of the n H_2O adsorbed surface system, E_{surf} is the total energy of the relaxed bare surface slab and $E_{\text{H}_2\text{O}}$ is the total energy of the relaxed water monomer in gas phase. Under this definition, E_{ad} is positive for all the systems studied. We decompose the interaction energy of the water dimer on surfaces into three parts: donor water-surface ($E_{\text{D-surf}}$), acceptor water-surface ($E_{\text{A-surf}}$), and water-water interactions ($E_{\text{water-water}}$) which predominately characterises the H-bond interaction at the adsorbate

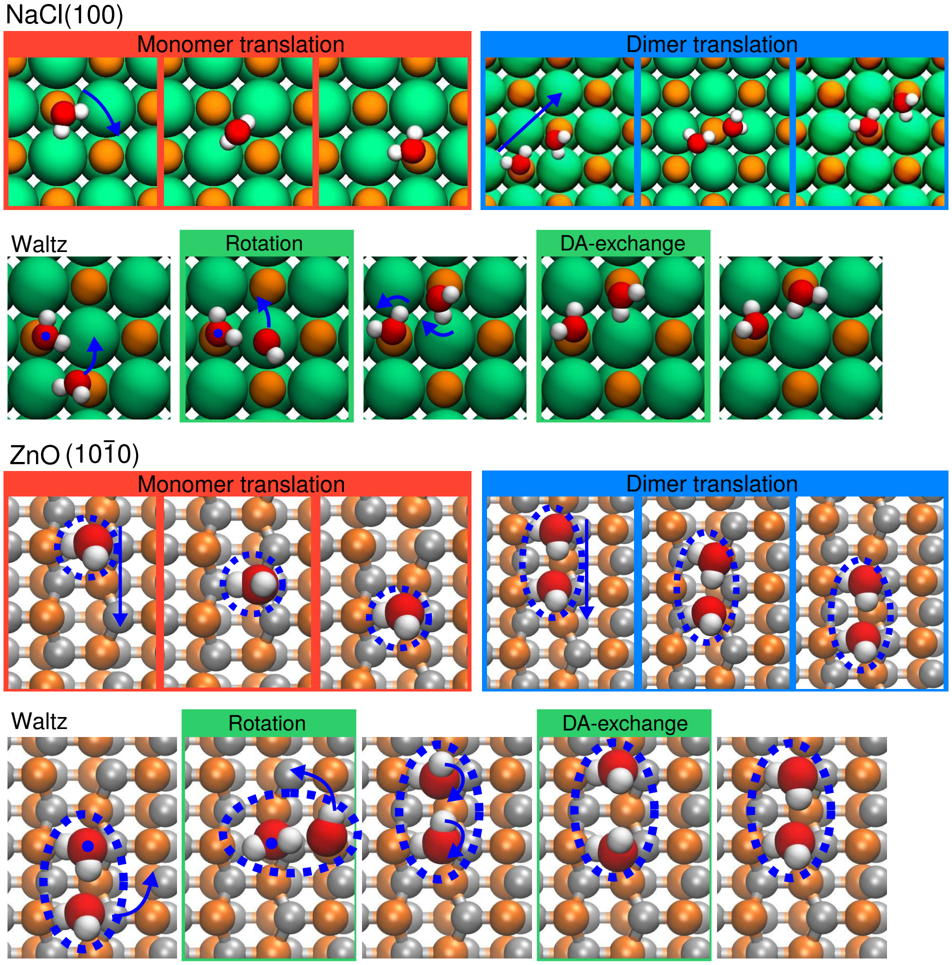

Supplementary Figure 2. Top views of water monomer, dimer translation, and dimer waltz diffusion pathways and transition state (TS) geometries on NaCl (100), and ZnO (10$\bar{1}$0) surfaces. For NaCl, orange: Na, green: Cl. For ZnO, orange: O, grey: Zn.

state. They are defined as the following:

$$\begin{aligned} E_{\text{water-water}} &= E_{\text{ad}}(\text{dimer}) - E_{\text{water-surf}} \\ E_{\text{water-surf}} &= E_{\text{ad}}(\text{donor}) + E_{\text{ad}}(\text{acceptor}), \end{aligned} \tag{2}$$

where $E_{\text{ad}}(\text{dimer})$ is the adsorption energy of a water dimer on the surface. $E_{\text{ad}}(\text{donor/acceptor})$ is the total energy of a water molecule on the surface fixed at the donor/acceptor geometry of the adsorbed water dimer.) We note that decompositions of adsorption energies are all to a certain extent arbitrary.

Supplementary Table 3 shows the decomposition results for the water dimer adsorption state (initial), and the diffusion transition states (TSs). Decomposition shows that on surfaces where the water-water interactions are smaller than or similar to that in the gas phase

surface	Initial			Dimer translation TS			Dimer DA exchange TS	
	$E_{\text{water-water}}$	$E_{\text{D-surf}}$	$E_{\text{A-surf}}$	$E_{\text{water-water}}$	$E_{\text{D-surf}}$	$E_{\text{A-surf}}$	$E_{\text{water-water}}$	$\frac{1}{2}E_{\text{water-surf}}$
Ag(111)	0.36	0.27	0.18	0.37	0.21	0.20	0.10	0.29
Cu(111)	0.41	0.29	0.20	0.43	0.18	0.20	0.08	0.32
Pt(111)	0.47	0.38	0.24	0.36	0.20	0.26	0.05	0.42
Pd(111)	0.43	0.44	0.25	0.41	0.26	0.29	0.06	0.45
Pd(100)	0.45	0.41	0.22	0.47	0.35	0.23	0.06	0.44
Rh(111)	0.44	0.50	0.27	0.44	0.25	0.29	0.04	0.53
Al(111)	0.49	0.41	0.25	0.36	0.25	0.26	0.03	0.44
Al(100)	0.59	0.34	0.20	0.53	0.12	0.21	0.03	0.39
NaCl(100)	0.16	0.42	0.41	0.21	0.17	0.26		
MgO(100)	0.17	0.53	0.54	0.17	0.34	0.43		
ZnO(10$\bar{1}$0)	0.15	0.93	0.97	0.18	0.41	0.44		

Supplementary Table 3. Decomposition of the interaction energies (units in eV) for water dimer diffusion on a variety of surfaces, which include the initial state (water dimer adsorbed on the top site), the dimer translational diffusion transition state (TS), and the water dimer DA exchange TS.

water dimer, both the H-bond donor and acceptor water bind to the surface equally strong. If the water-water interaction in the dimer is stronger than or similar to $E_{\text{D-surf}}$, the acceptor water interacts much weaker with the surface.

SUPPLEMENTARY NOTE 4. THE IMPACT OF SURFACE FLEXIBILITY ON WATER DIFFUSION ON NON-METAL SURFACES

We also computed the adsorption and diffusion of water monomers and dimers on flexible non-metal surfaces (Supplementary Table 4). We see that compared to fixed surfaces, using flexible surfaces quantitatively increase the water monomer and dimer diffusion barriers by 10%-30%. Yet the adsorption energies of water monomers and dimers are also increased on flexible surfaces, inline with the qualitative idea that the stronger the adsorbate sticks to the surface, the slower it diffuses. The only exception we see is water dimer diffusion on NaCl, where using a flexible surface stabilises the TS more than the initial state.

		E_{ad} (eV)		Diffusion barrier (eV)		$E_{water\text{-}water}$ (eV)	
		fixed surf	flex surf	fixed surf	flex surf	fixed surf	flex surf
NaCl	mono	0.45	0.48	0.15	0.17	-	-
	dimer	0.97	1.06	0.35	0.31	0.16	0.23
MgO	mono	0.54	0.60	0.12	0.16	-	-
	dimer	1.24	1.38	0.31	0.40	0.17	0.19
ZnO	mono	0.98	1.17	0.43	0.53	-	-
	dimer	2.05	2.41	1.00	1.10	0.15	0.15

Supplementary Table 4. Comparison of adsorption energies and diffusion barriers of water monomers and dimers on flexible and fixed surfaces. The H-bond energies for the water dimer obtained from the decomposition in Eq. 3 are also compared on flexible and fixed surfaces. Note that we only manage to converge the force of the TS of water dimer diffusion on flexible ZnO to 0.04 eV/Å.

One can also estimate the water-water interactions for water dimers on flexible surfaces via a more complicated decomposition. One viable treatment is to consider the surface deformation energy as part of the water-surface interactions in the decomposition in Eq. 2:

$$\begin{aligned} E_{\text{water-water}} &= E_{\text{ad}}(\text{dimer}) - E_{\text{water-surf}} \\ E_{\text{water-surf}} &= E_{\text{ad}}(\text{donor}) + E_{\text{ad}}(\text{acceptor}) - E_{\text{deform}}, \end{aligned} \tag{3}$$

in which $E_{\text{deform}} = E^*_{\text{surf}} - E_{\text{surf}}$ (E^*_{surf} is the energy of the substrate at the optimised geometry with the adsorbate). $E_{\text{D-surf}}$ and $E_{\text{A-surf}}$ are defined as same as in Eq. 2. We compared the water-water interactions for a water dimer on flexible and fixed surfaces, shown in Supplementary Table 4. One can see that using flexible surface also does not qualitatively change the water-water interaction in the decomposition. And finally, for the rotation step of the non-transitional water dimer diffusion on NaCl(100), using flexible surface changes the barrier by less than 0.01 eV (to 0.089 eV).

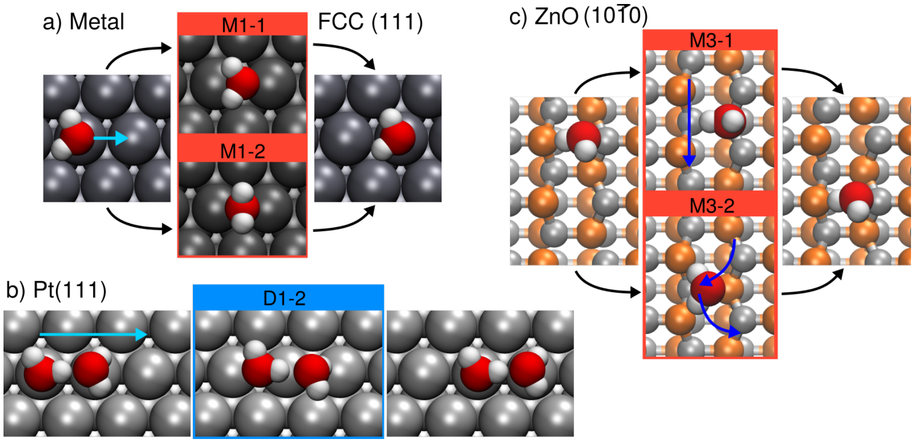

Supplementary Figure 3. Illustrations of the translational diffusion pathways that we have considered but not discussed in detail in the main text.

SUPPLEMENTARY NOTE 5. ADDITIONAL DISCUSSIONS ON THE WATER TRANSLATION DIFFUSION

We have also considered a few other monomer and dimer diffusion pathways. We showed in the main text that the water monomer has two diffusion pathways on transition metal (111) surfaces, here we discuss this in more detail. Almost flat monomer TSs are favoured on the (111) surfaces of Cu, Pt, and Pd, while the upright TSs are slightly higher in energies by 14, 21, and 12 meV on Cu, Pt, and Pd respectively. On Ag(111) and Rh(111), the flat position TSs cannot be found in the cNEB optimisations. For the dimer translational diffusion process on metals, we considered another pathway as shown in Supplementary Figure 3(b). This TS geometry is 50 meV higher in energy on Pt(111) compared to the TS discussed in the main text, and on Rh(111) the path in Supplementary Figure 3(b) is not found with cNEB optimizations, hence it is unlikely to be relevant. On ZnO(10$\bar{1}$0) we considered another water monomer diffusion TS (M3-2), shown in Supplementary Figure 3(c). This TS has the same energy (within 3 meV difference) as M3-1 on fixed substrate, and is less stable by 0.05 eV on flexible substrate, hence we only discuss M3-1 in the main text.

We have tested the correlation between the translational diffusion barrier and the adsorption energy, with two other definitions of the dimer adsorption energy:

$$E_{\mathrm{ad2}} = -(E_{n\mathrm{H_2O/surf}} - E_{\mathrm{surf}} - E_{\mathrm{nH_2O}}) \tag{4}$$

where $E_{\mathrm{nH_2O}}$ is the energy of a optimised gas phase water dimer. This means E_{ad2} is defined

with respect to a gas phase water dimer.

$$E_{\mathrm{ad3}} = -(E_{n\mathrm{H_2O/surf}} - E_{\mathrm{surf}} - E^*_{\mathrm{nH_2O}}) \tag{5}$$

where $E^*_{\mathrm{nH_2O}}$ is the energy of a gas phase water dimer fixed at the geometry on the surface.

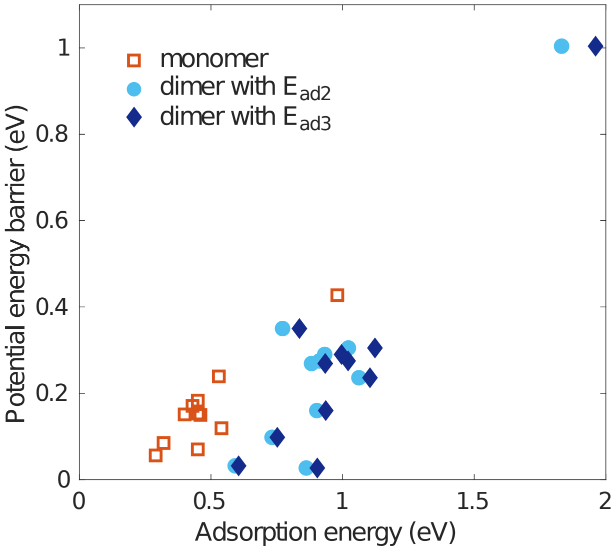

Supplementary Figure 4. Correlation between water monomer and dimer translational diffusion barriers and the adsorption energy with different definitions.

The results are shown in Supplementary Figure 4. The R^2 value of the linear regression for monomer and dimer with E_{ad2} is 0.68. Using E_{ad3} gives the same R^2 value. Despite that the correlation becomes better than with E_{ad}, none of the correlations are very good, and clearly worse than the correlation found in the main text.

SUPPLEMENTARY NOTE 6. ADDITIONAL DISCUSSIONS ON THE DIMER DA EXCHANGE DIFFUSION

The process of the two water exchanging their roles is the key step in the water dimer DA exchange diffusion mechanism proposed in ref.,[16] and there are many pathways for this DA exchange step. All of the different pathways can be identified by considering the possible permutations of the atoms in the water dimer,[17] and they can be categorised into three categories: "twist-twist" pathways, "twist-flip" pathways, and "flip-flip" pathways, as illustrated in Supplementary Figure 5. The rotation of a water molecule in the plane

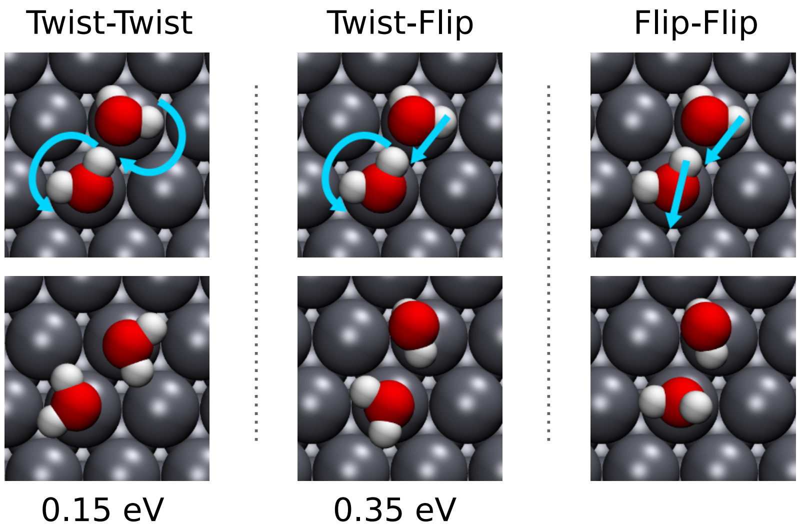

Supplementary Figure 5. Illustrations (on Pd(111) as an example) of the 3 categories of water DA exchange pathways. The top panels show the initial states (top view) and the blue arrows indicate the movement of the water molecules in the DA exchange pathways. The bottom panels show the TS geometries (top view) found for the DA exchange pathways. For the flip-flip pathway on a transition metal (111) surface, the bottom panel shows the initial guess of the TS, however, in the cNEB calculations the TS converged towards the twist-twist TS.

parallel to the surface is referred to as a "twist". The rotation of a water around one of its two O-H bonds is referred to as a "flip". In the twist-twist mechanism, the two water molecules rotate in the plane parallel to the surface, while the two O atoms switch height. For the twist-flip pathways, one water molecule performs a "twist", while the other water rotates around one of its two O-H bonds. The flip-flip pathways are mechanisms in which both waters perform a "flip" to rearrange the H-bond. Possible transition states (TSs) of the three categories are also shown in Supplementary Figure 5 (bottom panels). Depending on the type of the substrate, different categories of DA exchange paths can be favoured. On transition metal (111) surfaces, the twist-twist pathway is expected to have the lowest barrier, because a water molecule favours flat adsorption geometries on these surfaces. As an example, on Pd(111) the potential energy barrier of the twist-flip mechanism is 0.2 eV higher than that of the twist-twist mechanism. On ZnO($10\bar{1}0$) the flip-flip pathway is the most relevant.

Next we tested whether waltz diffusion barriers have and correlation with the adsorption

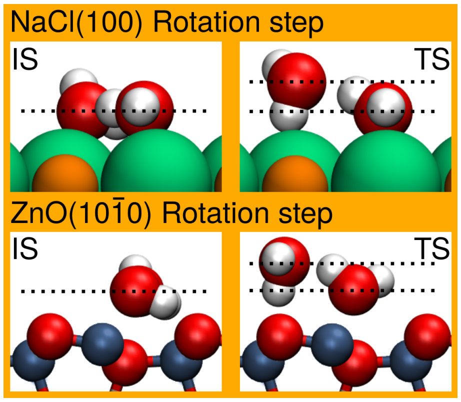

Supplementary Figure 6. Side view of rotation step on NaCl(100) and ZnO(10$\bar{1}$0), viewed from the [00$\bar{1}$] direction, showing height changes of the acceptor water at the TS. The atoms: green: Cl; orange: Na; dark blue: Zn.

energy. We used the definition of adsorption energy that gave the best correlation, which is E_{ad2}. The results are shown in Supplementary Figure 7, and the correlation becomes worse when the waltz diffusion barriers are added ($R^2 = 0.57$)

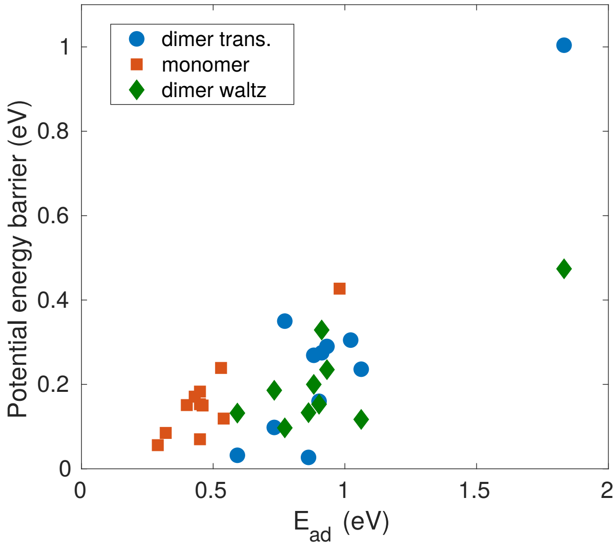

Supplementary Figure 7. Correlation between water monomer, dimer translational diffusion, and dimer waltz diffusion barriers and the adsorption energy (E_{ad2}).

SUPPLEMENTARY NOTE 7. WATER DIMER STEPWISE DIFFUSION AND DISSOCIATION ON METAL SURFACES

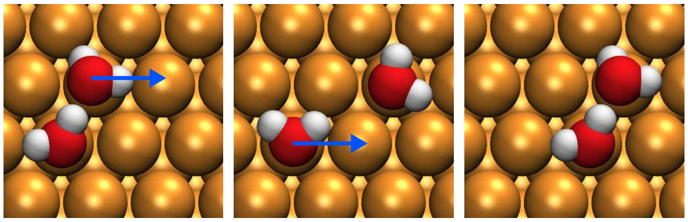

Supplementary Figure 8. The stepwise water dimer diffusion mechanism on Cu(111).

We considered a stepwise water dimer diffusion mechanism and tested it on Cu(111). The separated water dimer geometry (Supplementary Figure 8) is 0.07 eV higher in energy than the DA exchange TS on Cu(111), an amount close to the $E_{\text{water-water}}$ in Supplementary Table 3. This suggests that the stepwise diffusion mechanism is unfavourable compared to the DA exchange mechanism on Cu(111) and other transition metal (111) surfaces, as the DA exchange TS is further stabilised by the water-water interaction.

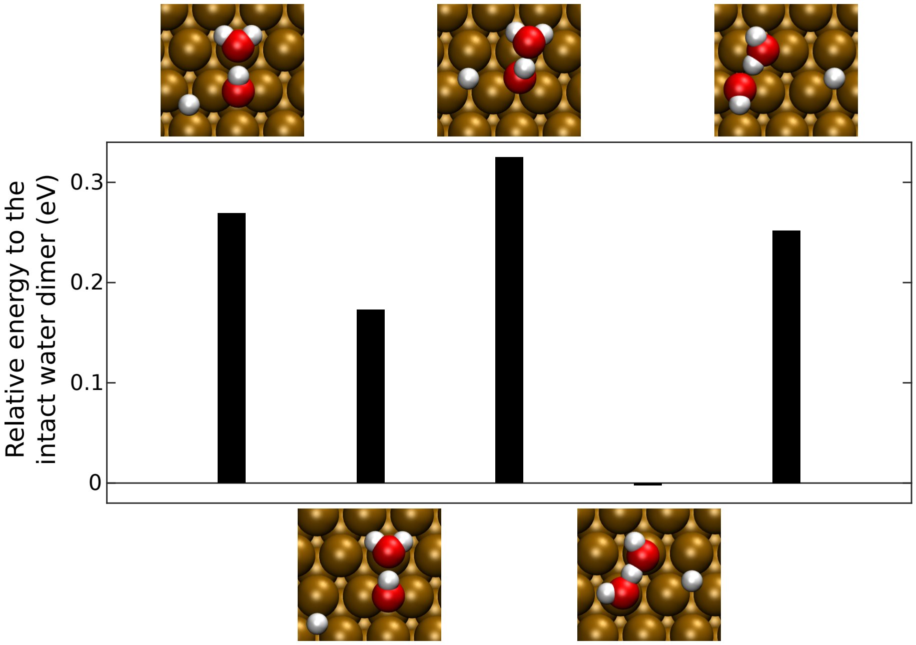

Supplementary Figure 9. Relative energies of dissociated water dimer geometries with respect to a intact water dimer on Rh(111).

Next we discuss whether water dissociation plays an important role for water diffusion on

surfaces. It has been shown that water monomers have high dissociation barriers (∼1 eV) on the surfaces we studied.[18,19] For the water dimer, we computed the energies of different dissociated geometries on Rh(111) as an example. As shown in Supplementary Figure 9, we see that among the different dissociated states, the hydrogen atom prefers the FCC site over the HCP site, and the hydroxyl-water complex prefers the top site over the FCC and bridge sites. Moreover, all the dissociated states considered have energies less stable or similar to the intact water dimer, hence we think an intact water dimer could stably exist on Rh(111), as well as on the other metal surfaces. We note that a water dimer favours dissociation on MgO(100) surface,[20] hence we do not thoroughly discuss water dimer diffusion on MgO(100) in the maintext.

SUPPLEMENTARY NOTE 8. INSTANTONS FOR WATER WITH CLASSICAL OXYGEN AND INSTANTONS FOR D_2O

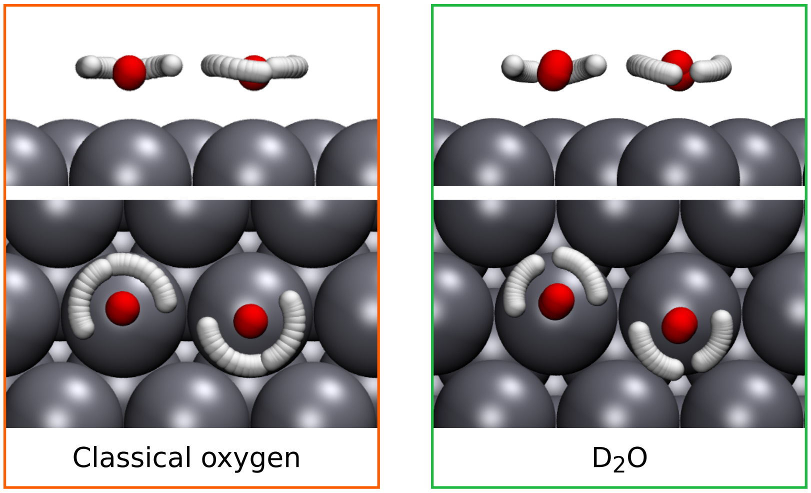

Supplementary Figure 10. Top and side views of the instantons optimised at 25 K for water with classical oxygen (left) and for D_2O (right).

We estimated the importance of O tunnelling in the water dimer at the deep tunnelling regime (25 K). Classical O was simulated by re-optimising the instanton with the mass of the oxygen atoms increased by ten fold. The instanton (Supplementary Figure 10) features no delocalisation of O atoms compared to the instanton with quantum O shown in the main text Supplementary Figure 4. Without O tunnelling, the instanton S action (see Eq. 2 in the

main text or Ref.[21]) is higher by what corresponds to a ∼40 fold slow-down in the tunnelling rate.

We also studied D_2O tunnelling at 25 K with the instanton rate theory (Supplementary Figure 10), in a (smaller) 3×3 unit cell. The results show that tunnelling is less significant for D_2O water, only reducing the classical barrier by 17 meV at 25 K, compared to 38 meV for H_2O at 25 K using the same unit cell.

SUPPLEMENTARY NOTE 9. SENSITIVITY OF THE DIFFUSION RATES TO THE POTENTIAL ENERGY BARRIERS

Of course the DFT barriers are subject to uncertainties, hence here we discuss the sensitivity of the diffusion rates and the temperature at which dimer diffusion becomes faster with respect to the uncertainties in the potential energy barriers of water diffusion on Pd(111). As shown in Supplementary Figure 11, if the monomer and dimer DA exchange diffusion

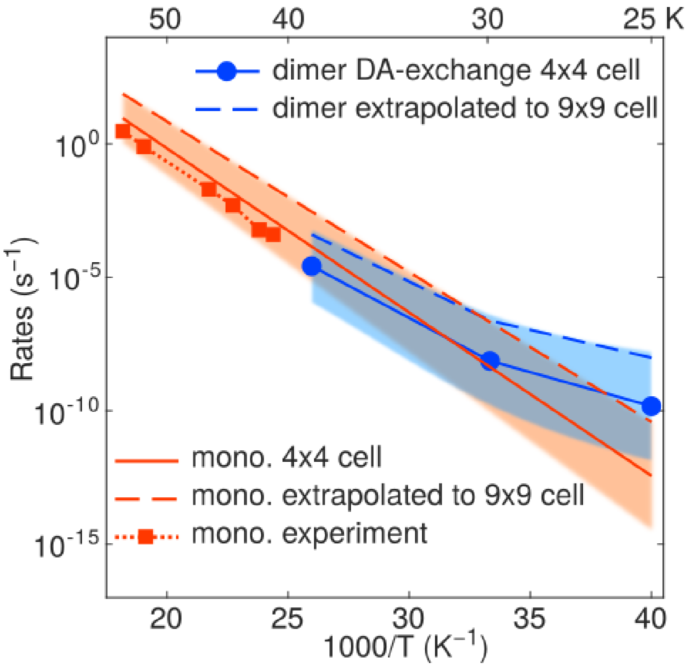

Supplementary Figure 11. Sensitivity of the diffusion rates to the potential energy barriers for the water monomer and dimer DA exchange diffusion processes. The coloured area shows the change of the diffusion rates with a barrier uncertainty of ± 10 meV. The dashed lines show the rates extrapolated to the 9 × 9 cell using the barrier difference between the 4 × 4 and 9 × 9 cell given in Supplementary Figure 1. Experimental rates from ref.[9] are shown for comparison.

rates are extrapolated to the 9×9 cell using the barrier difference between the 4×4 and 9×9 cell given in Supplementary Figure 1, the rates increase by 1-2 orders of magnitude. Yet since both the monomer and dimer barriers increase by a similar amount ($\sim$10 meV), the temperature at which the dimer diffusion becomes faster is barely changed by this extrapolation. Furthermore, we considered the impact of a general 10 meV uncertainty on the diffusion rates, as shown by the coloured areas in Supplementary Figure 11. We still see the trend that the dimer diffusion tends to become faster than monomer diffusion as the temperature decreases. However, if the uncertainties make the dimer DA exchange barrier larger than monomer diffusion barrier, i.e. more than 30 meV barrier reduction from tunnelling at 25 K, then dimer diffusion will likely become faster than monomer diffusion at below 25 K instead.

SUPPLEMENTARY NOTE 10. THE IMPACT OF ELECTRIC FIELD AND ROTOR PARTITION FUNCTION

In the STM experiment on Pd,[9] a bias voltage (on the order of 0.1 V) was applied between the substrate and the STM tip. This results in a weak electric field (on the order of 0.01 V/Å) which the water molecules on the surface can experience. With this in mind, cNEB calculations with external electric fields applied perpendicular to the slab were performed. The results, shown in Supplementary Figure 5, show that even with electric fields of 0.05

E field (V/Å)	Water monomer	Dimer translation	Dimer DA exchange
0.05	0.148	0.163	0.162
0	0.150	0.160	0.153
-0.05	0.153	0.155	0.142

Supplementary Table 5. Dependence of the water diffusion barriers (units in eV) on Pd(111) with respect to the external electric field applied perpendicular to the surface.

V/Å, their effects on the water diffusion barriers are insignificant. The dimer diffusion TSs barely change, while the tilt angle of the water monomer in the TS can be affected by the weak electric field.

The contribution of the anharmonic effects of the water dimer rotation around the axis

perpendicular to the surface (see Supplementary Figure 1(c) in the maintext) has also been considered. The rotation barrier, for example, on Pd(111) is ∼9 meV (calculated with cNEB), which is larger than $k_B T$ in the temperature range considered (25 − 40 K). We calculated the hindered rotor partition function[22] in this temperature range, and found that it is only twice as large as the harmonic approximation (the harmonic frequency for this mode is ∼20 cm^{-1} on Pd(111)). Even in the free rotor limit, the rotational partition function will only contribute no more than a factor of 5 to the reduction of the water dimer diffusion rate prefactor. The water monomer also has a rotation mode around the axis perpendicular to the surface (∼40 cm^{-1} on Pd(111)), in which the free rotor partition function is within a factor of 2 with the harmonic approximation at 25 − 40 K. Hence, the anharmonic rotational partition functions do not have a significant impact on the diffusion rate here, which is dominated by the exponential term.

SUPPLEMENTARY REFERENCES

[1] J. Klimeš, D. R. Bowler, and A. Michaelides, Phys. Rev. B **83**, 195131 (2011).

[2] Y. Zhang and W. Yang, Phys. Rev. Lett. **80**, 890 (1998).

[3] S. Grimme, S. Ehrlich, and L. Goerigk, J. Comput. Chem. **32**, 1456 (2011).

[4] J. Tao, J. P. Perdew, V. N. Staroverov, and G. E. Scuseria, Phys. Rev. Lett. **91**, 146401 (2003).

[5] J. Klimeš, D. R. Bowler, and A. Michaelides, J. Phys. Condens. Matter **22**, 022201 (2010).

[6] M. J. Gillan, D. Alfè, and A. Michaelides, J. Chem. Phys. **144**, 130901 (2016).

[7] J. G. Brandenburg, J. E. Bates, J. Sun, and J. P. Perdew, Phys. Rev. B **94**, 115144 (2016).

[8] L. Goerigk, A. Hansen, C. Bauer, S. Ehrlich, A. Najibi, and S. Grimme, Phys. Chem. Chem. Phys. **19**, 32147 (2017).

[9] T. Mitsui, M. K. Rose, E. Fomin, D. F. Ogletree, and M. Salmeron, Science **297**, 1850 (2002).

[10] A. V. Krukau, O. A. Vydrov, A. F. Izmaylov, and G. E. Scuseria, J. Chem. Phys. **125**, 224106 (2006).

[11] S.-C. Heidorn, C. Bertram, P. Cabrera-Sanfelix, and K. Morgenstern, ACS Nano **9**, 3572 (2015).

[12]C. Bertram, W. Fang, P. Pedevilla, A. Michaelides, and K. Morgenstern, Nano Lett. **19**, 3049 (2019).

[13]S. Grimme, A. Hansen, J. G. Brandenburg, and C. Bannwarth, Chem. Rev. **116**, 5105 (2016).

[14]B. Li, A. Michaelides, and M. Scheffler, Surf. Sci. **602**, L135 (2008).

[15]J. Matthiesen, J. O. Hansen, S. Wendt, E. Lira, R. Schaub, E. Lægsgaard, F. Besenbacher, and B. Hammer, Phys. Rev. Lett. **102**, 226101 (2009).

[16]V. A. Ranea, A. Michaelides, R. Ramírez, P. L. de Andres, J. A. Vergés, and D. A. King, Phys. Rev. Lett. **92**, 136104 (2004).

[17]J. O. Richardson, C. Pérez, S. Lobsiger, A. A. Reid, B. Temelso, G. C. Shields, Z. Kisiel, D. J. Wales, B. H. Pate, and S. C. Althorpe, Science **351**, 1310 (2016).

[18]M. Pozzo, G. Carlini, R. Rosei, and D. Alfè, J. Chem. Phys. **126**, 164706 (2007).

[19]A. A. Phatak, W. N. Delgass, F. H. Ribeiro, and W. F. Schneider, J. Phys. Chem. C **113**, 7269 (2009).

[20]X. L. Hu, J. Carrasco, J. Klimeš, and A. Michaelides, Phys. Chem. Chem. Phys. **13**, 12447 (2011).

[21]J. O. Richardson and S. C. Althorpe, J. Chem. Phys. **131**, 214106 (2009).

[22]L. H. Sprowl, C. T. Campbell, and L. Árnadóttir, J. Phys. Chem. C **120**, 9719 (2016).
